# CabHLH79 Acts Upstream of *CaNAC035* to Regulate Cold Stress in Pepper

**DOI:** 10.3390/ijms23052537

**Published:** 2022-02-25

**Authors:** Ziyu Wang, Yumeng Zhang, Huifang Hu, Lang Chen, Huafeng Zhang, Rugang Chen

**Affiliations:** 1 College of Horticulture, Northwest A&F University, Yangling 712100, China; wangziyv@nwafu.edu.cn (Z.W.); Kexuanzhangyumeng@163.com (Y.Z.); huifang@nwafu.edu.cn (H.H.); chenlang@nwafu.edu.cn (L.C.); huafengzhang@nwafu.edu.cn (H.Z.); 2 Shaanxi Engineering Research Center for Vegetables, Yangling 712100, China

**Keywords:** *CabHLH79*, bHLH transcription factor, reactive oxygen species, cold stress

## Abstract

Cold stress is one of the main restricting factors affecting plant growth and agricultural production. Complex cold signaling pathways induce the expression of hundreds of cold-sensitive genes. The NAC transcription factor *CaNAC035* has previously been reported to significantly influence the response of pepper to cold stress. Here, using Yeast one-hybrid (Y1H) library screened to search for other relevant molecular factors, we identified that *CabHLH79* directly binds to the *CaNAC035* promoter. Different basic helix–loop–helix (bHLH) transcription factors (TFs) in plants significantly respond to multiple plant stresses, but the mechanism of bHLHs in the cold tolerance of pepper is still unclear. This study investigated the functional characterization of *CabHLH79* in the regulation of cold resistance in pepper. Down-regulation of *CabHLH79* in pepper by virus-induced gene silencing (VIGS) increased its sensitivity to low temperature, whereas overexpression of *CabHLH79* in pepper or *Arabidopsis* enhanced cold resistance. Compared with control plants, VIGS mediated of *CabHLH79* had lower enzyme activity and related gene expression levels, accompanied by higher reactive oxygen species (ROS) accumulation, relative electrolyte leakage (REL), and malondialdehyde accumulation (MDA) contents. Transient overexpression of *CabHLH79* pepper positively regulated cold stress response genes and ROS genes, which reduced REL and MDA contents. Similarly, ectopic expression of *CabHLH79* in *Arabidopsis* showed less ROS accumulation, and higher enzymes activities and expression levels. These results indicated that *CabHLH79* enhanced cold tolerance by enhancing the expression of ROS-related and other cold stress tolerance-related genes. Taken together, our results showed a multifaceted module of bHLH79-NAC035 in the cold stress of pepper.

## 1. Introduction

Cold stress has always been an extreme external environmental factor that deeply affects plant growth and crop yield, and even leads to plant death [1,2]. Cold stress can affect the physiological response, germination, accelerated senescence, oxidative damage, membrane damage, and tissue destruction of plants [3,4]. Therefore, plants have also evolved a set of complex mechanisms to resist harsh environmental stresses [5,6,7]. Stress responses are known to include a variety of signaling pathways that form complex networks of structural and regulatory proteins encoded by different genes that play a direct or indirect role in protecting plants from abiotic stresses [8,9,10,11]. Transcription factors (TFs) are important regulatory proteins, which control the expression of target genes by binding to specific cis-acting elements in promoters [12]. Therefore, identification and characterization of TFs that respond to stress are crucial to elucidate the molecular network associated with stress response [13]. Cold stress can induce the expression of multiple TFs, including C-repeat-binding factor (CBF), APETALA2 (AP2), basic region/leucine zipper (bZIP), basic helix–loop–helix (bHLH), MYB, and NAC families, which can bind to the promoter of stress-related genes and regulates their expression [14,15,16,17]. The bHLH TFs are the second largest family of plant-specific transcription factors after the MYBs. They are widely distributed in plants and play a key role in coping with adverse environments such as cold, salt, drought, and osmotic stress. The bHLHs family consists of about 60 amino acids and has two functional domains, namely a basic domain and a HLH domain [18,19].And, bHLH TFs can bind to the E-box (5′-CANNTG-3′) or the G-box (5′-CACGTG-3′) element to perform its function [20].

The bHLH transcription factors have been identified in many plants, such as *Arabidopsis*, rice, *Brachypodium distachyon*, wheat, maize, *Brassica napus*, and pepper [21,22,23,24,25,26,27]. Many bHLH transcription factors have been reported to participate in various stresses (e.g., salt, drought, and cold stress) and play important roles in biotic and abiotic stress responses. For instance, *NtbHLH123* enhances tolerance to cold stress by modulating reactive oxygen species homeostasis in tobacco [28]. Overexpression of *PtrbHLH* confers cold tolerance in pummelo by modulation of H_2_O_2_ level via regulating a CAT gene [29]. *Arabidopsis AtMYC2* contributes to salt tolerance by directly regulating proline biosynthesis [30]. The bHLH transcription factor *SbbHLH85* of sorghum in modulation of salt tolerance by modulating root hair growth [31]. Wheat bHLH transcription factor gene *TabHLH1* regulates osmotic tolerance through modulation of the ABA-dependent pathway [32]. Overexpression of *OrbHLH001* enhances the tolerance to freezing and salt stresses in transgenic *Arabidopsis* [33]. *AhbHLH112* in peanut positively regulates drought tolerance [34]. *MfbHLH38* plays a positive regulatory role in responding to drought and salinity stresses in *Myrothamnus flabellifolia* [35]. Overexpression of *OrbHLH2* can enhance salt tolerance [36]. In wheat, *TabHLH49* can regulate drought tolerance [37]. The MYB transcription factor *MdMYB308L* in apples interacts with *MdbHLH33* positively regulates cold tolerance and anthocyanin accumulation [38]. In addition, *AtbHLH115* plays a key role in the maintenance of Fe homeostasis in *Arabidopsis* [39]. *OsbHLH6* confers disease resistance by regulating SA and JA signaling in rice [40]. Overexpression of the *SlbHLH22* gene revealed that it is highly involved in controlling flowering time and promoting fruit ripening and improved carotenoid accumulation [41]. Although bHLH TFs have been analyzed and characterized in many plants, the function of the bHLH genes in pepper remains unclear.

35S:CabHLH79-GFP vector was overexpressed in pepper leaves to explore the function of *CabHLH79* in response to cold. Molecular breeding is one of the most efficient ways of breeding cold-tolerant varieties. When plants respond to cold and drought stress, *bHLH7/bHLH43/bHLH79/bHLH93* genes in the bHLH family are significantly up-regulated [42]. To date, 122 bHLH transcription factors have been found in pepper [27]. However, only a few bHLH transcription factors have been identified in pepper for their functions. The regulatory mechanism and biological function of many CabHLH proteins in peppers are still unclear. In our previous study, we demonstrated that *CaNAC035* played an important role in plant response to abiotic stress, including cold stress [43]. In this study, we identified *CabHLH79* (Capana03g001053) as the upstream factor of *CaNAC035* (Capana05g000569) in pepper through Yeast one-hybrid (Y1H) assay. Sequence analysis revealed that *CabHLH79* had the same conserved domains as bHLH transcription factors. Phylogenetic analysis indicated that *CabHLH79* had high homology with potato, tomato, and tobacco. Many previous studies have found that abiotic stress can induce the expression of many bHLH TFs with different functions in plants. Therefore, we speculated that *CabHLH79* may play an essential role in regulating various processes. This study provides valuable information for further analysis of physiological and biochemical characteristics of bHLH transcription factors in pepper and other plants.

## 2. Results

### 2.1. CabHLH79 Directly Targets the CaNAC035 Promoter

**Figure 1:**
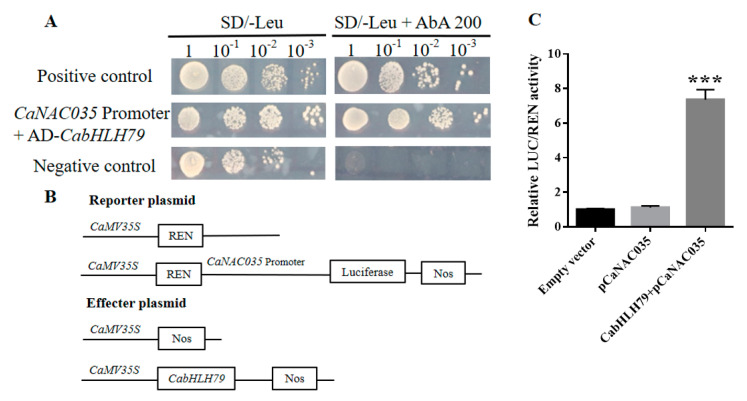
*CabHLH79* binds to and activates the *CaNAC035* promoter. (**A**) Yeast one-hybrid (Y1H) assay on binding of *CabHLH79* directly to the promoter region of *CaNAC035*. *CaNAC035* promoter + AD−CabHLH79, insert the *CaNAC035* promoter truncated segment (1−520 bp) into the pAbAi vector as a bait, and *CabHLH79* was inserted into the pGADT7 vector as prey. pGADT7−p53+pAbAi−p53 was a positive control and pGADT7 + CaNAC035 was a negative control. Yeast cells were grown on SD/−Leu plates with 200 ng/ mL Aureobasidin A(AbA). (**B**) Schematic representation of the firefly luciferase (LUC) reporter vector containing the *CaNAC035* promoter and the effector vectors expressing *CabHLH79* under the control of the 35S promoters. The open reading frames of *CabHLH79* were fused to a pGreenII 62−SK vector. The promoter sequence of *CaNAC035* was cloned into a pGreenII 0800−LUC vector. (**C**) LUC/Renillaluciferase (REN) activities detected from the reporter system described in (**A**), testing the effects of *CabHLH79* on the expression of *CaNAC035*. Empty vector, pGreenII 62-SK + pGreenII 0800−LUC; pCaNAC035, pGreenII 62−SK+promoterCaNAC035−pGreenII 0800−LUC; *CabHLH79*+pCaNAC035, *CabHLH79*−pGreenII 62−SK+promoterCaNAC035−pGreenII 0800−LUC. All data of three independent biological replicates were expressed by Means ± SDs. * representing significant difference (*** *p* < 0.001).

Our previous studies have found that *CaNAC035* plays important role in the response to abiotic stress [43]. To investigate the upstream regulator of *CaNAC035*, the promoter fragment of *CaNAC035* was used to screen the pepper cDNA library. We identified a total of ten positive colonies that may interact with the *CaNAC035* promoter, and four of them were TF (Table S2). To confirm the interaction between *CabHLH79* and *CaNAC035* promoter, Y1H detection was performed. The full length of *CabHLH79* was used as the prey, the *CaNAC035* promoter fragment was used as bait. All Y1H yeast strains grow normally on SD/-Leu medium but only the positive control and pAbAi−*CaNAC035*+AD−*CabHLH79* survived on the SD/-Leu medium with 200 ng/mL AbA (Figure 1A). These results showed that *CabHLH79* can bind to the *CaNAC035* promoter. Subsequently, to investigate the ability of *CabHLH79* to activate the *CaNAC035* gene, a dual-luciferase (LUC) assay was performed using *CabHLH79* as an effector in tobacco (Figure 1B). We found that co-transformation of effector factor and reporter gene significantly increased promoter activity, as indicated by the LUC/REN ratio (Figure 1C). These results indicated that *CabHLH79* can activate the *CaNAC035* promoter.

### 2.2. Characterization of CabHLH79 and Bioinformatics Analysis

The CDS region of *CabHLH79* is 834 bp, which encodes 277 amino acids, has an isoelectric point of 5.40, and a predicted molecular weight of 29.93 kDa. *CabHLH79* belongs to the MYC-type bHLH transcription factor family because it contains a conserved bHLH domain. MEGAX software is used by us to construct a phylogenetic tree and analyze the evolutionary relationship between *CabHLH79* and bHLH proteins of different species such as *Arabidopsis*, tobacco, potato, tomato, and cucumber. Phylogenetic analysis showed that *CabHLH79* had high homology with potato, tomato, and tobacco (Figure S1B). To further analyze the structure of *CabHLH79*, DNAMAN software was used to compare the amino acid sequences of *CabHLH79* with other species (Figure S1A).

### 2.3. Silencing of CabHLH79 Decreases Tolerance to Cold Stress in Pepper

**Figure 2:**
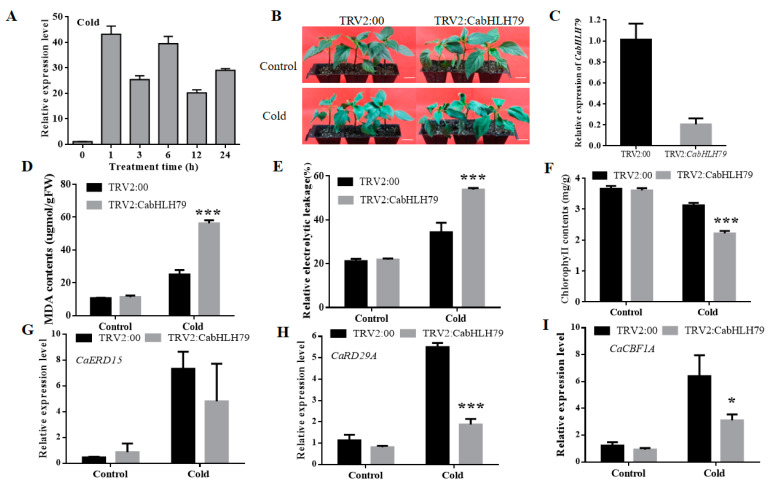
The measurement of physiological indices of *CabHLH79*-gene-silenced pepper plants under cold stress. (**A**) The expression level of *CabHLH79* under cold stress. (**B**) The phenotype of pepper plants with reduced expression of *CabHLH79* under cold stress used virus-induced gene silencing (VIGS). The white line is used as a scale bar (length 2 cm). (**C**) The silencing efficiency of *CabHLH79*. (**D**–**F**) malondialdehyde accumulation (MDA), relative electrolyte leakage (REL), chlorophyll contents. (**G**–**I**) RT-qPCR analysis of *CaERD15, CaRD29A, CaCBF1A.* All data of three independent biological replicates were expressed by Means ± SDs. * representing significant difference (* *p* < 0.05, *** *p* < 0.001).

In order to determine the expression patterns of *CabHLH79*, we performed RT-qPCR assays on RNA extracted from the *C. annuum* cultivar P70 plant. The results showed that *CabHLH79* was strongly induced by cold stress. The mRNA level of *CabHLH79* was expressed in a large amount in 1–24 h, and the expression level was up to 40 folds in 1 h (Figure 2A). It was worth noting that *CabHLH79* exhibited high expression levels under cold stress, which clarified that *CabHLH79* may be a positive regulator in response to cold stress. To investigate the function of *CabHLH79* under cold stress, *CabHLH79-*silenced and control plants were exposed to cold stress (4 °C) for 3 days. *CabHLH79-*silenced plants showed significantly severe leaf damage symptoms (Figure 2B), which indicated that the *CabHLH79-*silenced plants were less resistant to cold stress. The silencing efficiency was measured through RT-qPCR, which was almost 85% (Figure 2C).

To confirm the silencing of the *CabHLH79* response to cold stress, we measuredmalondialdehyde accumulation (MDA), relative electrolyte leakage (REL), and chlorophyll contents. After exposure to cold stress, MDA and RELcontents in *CabHLH79-*silenced plants were higher than the control plants (Figure 2D,E), which means that the *CabHLH79-*silenced plants have more severe cell membrane per-oxidation than the control plants. At the same time, the chlorophyll contents in the *CabHLH79-*silenced pepper were lower than control plants (Figure 2F), revealing that the silenced plants were more damaged under cold stress. Furthermore, the expressions of cold-related genes (*CaERD15, CaRD29A, CaCBF1A*) were determined by RT-qPCR (Figure 2G–I). The data showed that the expression of the *CaERD15, CaRD29A, CaCBF1A* were significantly higher in TRV2 plants than *CabHLH79*-silenced pepper plants. In summary, the silencing of *CabHLH79* reduced tolerance to cold stress.

### 2.4. Silencing of CabHLH79 in Pepper Cause Excessive Accumulation of ROS

**Figure 3:**
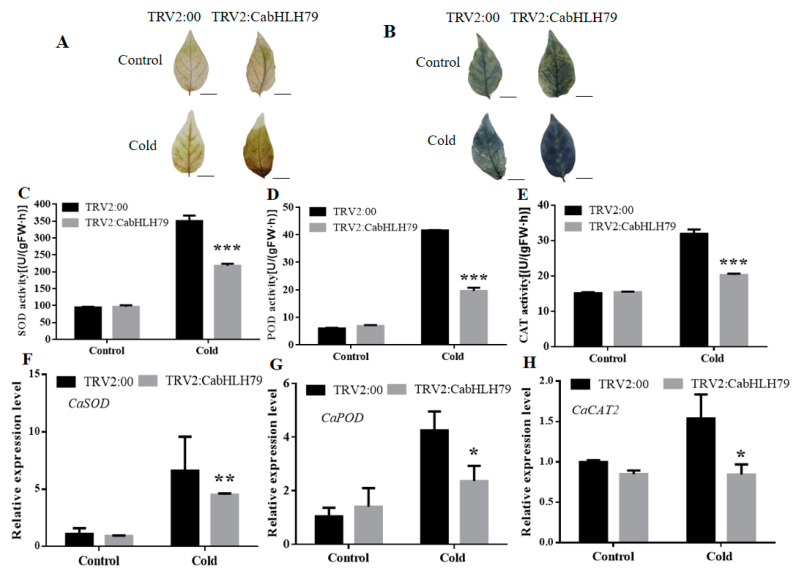
Reactive oxygen species (ROS) levels in *CabHLH79*-silenced and control plants. (**A**) DAB staining. (**B**) NBT staining. The black line is used as a scale bar (length 1 cm). (**C**) SOD activity. (**D**) POD activity. (**E**) CAT activity. (**F**–**H**) RT-qPCR analysis of *CaSOD*, *CaPOD*, and *CaCAT2*. All data of three independent biological replicates were expressed by Means ± SDs. * representing significant difference (* *p* < 0.05, ** *p* < 0.01, *** *p* < 0.001).

We also performed the DAB and NBT staining (Figure 3A,B), the results showed that the *CabHLH79*-silenced plants were darker after staining, indicating that more H_2_O_2_ and O_2_^•−^ contents were accumulated in *CabHLH79*-silenced plants. Additionally, we measured the activities of the main ROS-scavenging enzymes (CAT, SOD, and POD). The activities of CAT, SOD, and POD in silenced-*CabHLH79* plants were lower in the control plants (Figure 3C–E). The positive effect of *CabHLH79* on antioxidant enzyme activity suggested that *CabHLH79* might be involved in the regulation of reactive oxygen species (ROS)homeostasis under cold stress. Therefore, we detected the expression levels of ROS-related genes in silenced-*CabHLH79* and control plants under cold treatment. The results showed that knockdown of *CabHLH79* decreased the expression levels of ROS-related genes (*CaCAT2*, *CaSOD,* and *CaPOD*) (Figure 3F–H). These results suggest that *CabHLH79* may be a key upstream regulator of some ROS-related genes.

### 2.5. Transient Overexpression of CabHLH79 in Pepper Enhances Cold Stress Tolerance

**Figure 4:**
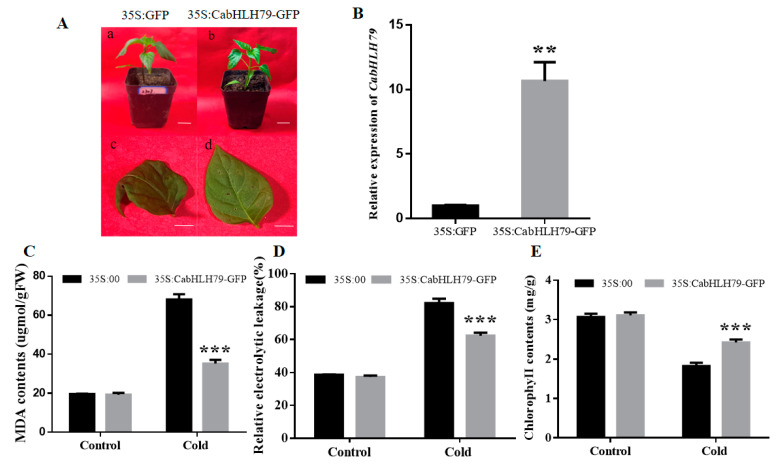
The measurement of physiological indices of *CabHLH79-*TO pepper plants under cold stress. (**A**) The transient expression of *CabHLH79* gene alleviates the damage of pepper plants to low temperature. The white line in a, b is used as a scale bar (length 1.75 cm). The white line in c, d is used as a scale bar (length 0.5 cm). (**B**) The expression level of *CabHLH79.* (**C**–**E**) MDA, REL, chlorophyll contents. All data of three independent biological replicates were expressed by Means ± SDs. * representing significant difference (** *p* < 0.01, *** *p* < 0.001).

35S:CabHLH79-GFP vector was overexpressed in pepper leaves to explore the function of *CabHLH79* in response to cold stress. We detected the transcription level of *CabHLH79* transient overexpression (TO) plants by RT-qPCR. It was found that the expression level of *CabHLH79* in *CabHLH79-*TO pepper was 10 folds higher than that of control (Figure 4B). To detect the effect of *CabHLH79-*TO in response to cold stress, *CabHLH79-*TO and control plants were exposed to cold stress (4 °C) for 3 days. The control plants showed significantly severe leaf damage symptoms (Figure 4A). Moreover, we measured MDA, REL, and chlorophyll contents. After exposure to cold stress, MDA and RELin *CabHLH79-*TO plants were lower than the control plants (Figure 4C,D). On the contrary, the chlorophyll content of *CabHLH79-*TO plants was higher than that of control plants (Figure 4E), indicating that transient overexpression of *CabHLH79* in pepper enhanced the cold resistance.

**Figure 5:**
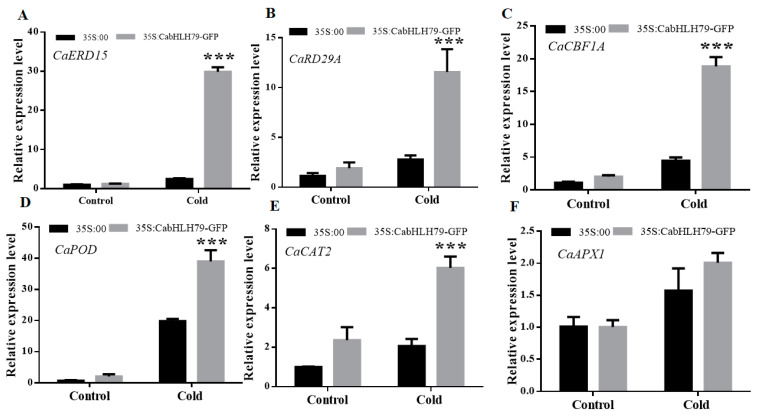
Transcription levels of cold-related genes and ROS-related genes. (**A**–**F**) RT-qPCR analysis of *CaERD15*, *CaRD29A*, *CaCBF1A*, *CaPOD*, *CaCAT2*, *CaAPX1*. All data of three independent biological replicates were expressed by Means ± SDs. * representing significant difference (*** *p* < 0.001).

Furthermore, the expression of cold-related genes and ROS-related genes (*CaERD15*, *CaRD29A*, *CaCBF1A*, *CaPOD*, *CaCAT2*, and *CaAPX1*) was determined by RT-qPCR (Figure 5). After cold stress, the transcript levels of *CaERD15*, *CaRD29A*, *CaCBF1A*, *CaPOD*, *CaCAT2,* and *CaAPX1* in *CabHLH79-*TO plants were significantly higher than those in control plants. The data indicated that transient overexpression of *CabHLH79* in pepper enhanced cold stress tolerance.

### 2.6. Overexpression of CabHLH79 in Arabidopsis Enhances Tolerance to Cold Stress

**Figure 6:**
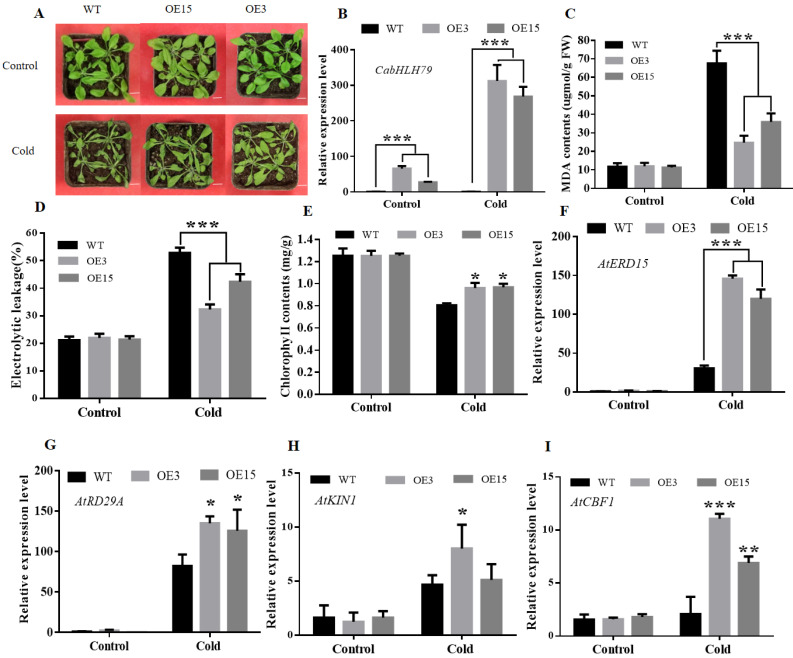
Overexpression of *CabHLH79* in *Arabidopsis* improves tolerance to low temperature stress. (**A**) The phenotype of *CabHLH79* overexpression and WT line under cold stress. The white line is used as a scale bar (length 1 cm). (**B**) Transcriptional levels of *CabHLH79* under cold stress. (**C**–**E**) MDA, REL, and Chlorophyll contents. (**F**–**I**) RT-qPCR analysis of *AtERD15*, *AtRD29A, AtKIN1,* and *AtCBF1*. All data of three independent biological replicates were expressed by Means ± SDs. * representing significant difference (* *p* < 0.05, ** *p* < 0.01, *** *p* < 0.001).

To investigate the role of *CabHLH79* overexpression in *Arabidopsis* under low temperature stress, 4-week-old *Arabidopsis* were treated at 4 °C for 3 days. WT plants showed more severe wilting than transgenic plants, suggesting that *CabHLH79* may be involved in cold resistance (Figure 6A). We detected the transcription level of *CabHLH79* transgenic lines by RT-qPCR. It was found that the expression of *CabHLH79* transgenic lines in *Arabidopsis* was higher than WT under cold stress (Figure 6B). Subsequently, we tested the MDA, REL, and chlorophyll contents of WT and transgenic lines. Before stress, the MDA, REL, and chlorophyll contents of WT and transgenic lines were no obvious differences. However, after cold treatment, the MDA and REL levels of the transgenic lines were significantly lower than WT (Figure 6C,D), the chlorophyll content was significantly higher than WT (Figure 6E). In order to further explore the mechanism of *CabHLH79*, RT-qPCR was used to analyze the expression of cold-related genes *AtERD15*, *AtRD29A, AtKIN1,* and *AtCBF1*. These data indicated that when plants were subjected to cold stress, cold-related genes were significantly increased in transgenic lines (Figure 6F–I). In summary, our results indicated that overexpression of *CabHLH79* in *Arabidopsis* significantly improved cold tolerance.

### 2.7. The Higher Enzymes Activities and Expression Levels of CabHLH79 Transgenic Arabidopsis

**Figure 7:**
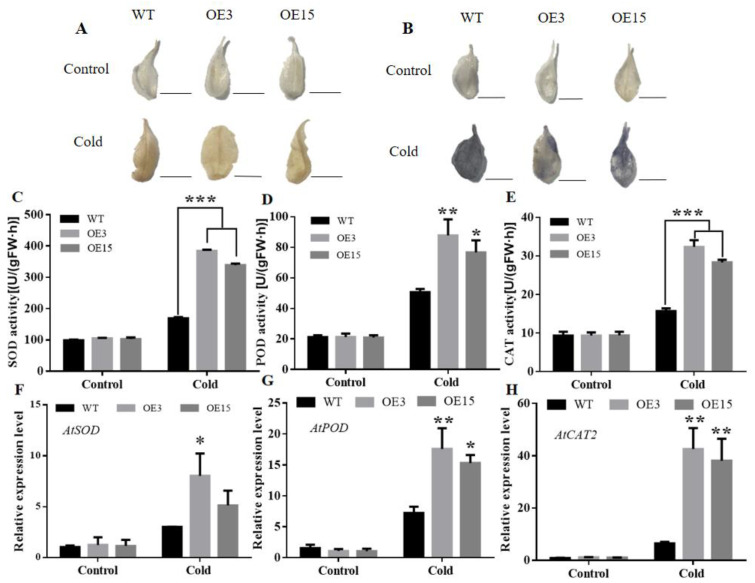
Reactive oxygen species (ROS) levels in WT and transgenic *Arabidopsis* under cold stress. (**A**) DAB staining. (**B**) NBT staining. The black line is used as a scale bar (length 0.8 cm). (**C**) SOD activity. (**D**) POD activity. (**E**) CAT activity. (**F**–**H**) RT-qPCR analysis of *AtSOD*, *AtPOD,* and *AtCAT2*. All data of three independent biological replicates were expressed by Means ± SDs. * representing significant difference (* *p* < 0.05, ** *p* < 0.01, *** *p* < 0.001).

When plants are subjected to cold stress, many physiological damages are caused, including the accumulation of ROS. Antioxidant enzymes play an important role in ROS detoxification and promote ROS scavenging under abiotic stress [44,45]. After cold stress, the DAB and NBT staining of the transgenic plant leaves appeared less blue or brown colors than WT (Figure 7A,B). After cold treatment, the three enzymatic activities of POD, SOD, and CAT in transgenic plants were significantly higher than WT (Figure 7C–E), which was consistent with the lower ROS accumulation. In order to further understand the molecular mechanism of *CabHLH79* overexpression enhancing cold tolerance, RT-qPCR was used to analyze the mRNA expression levels of antioxidant genes *AtSOD*, *AtPOD*, and *AtCAT2* in WT and transgenic lines. The transcription levels of these three tested genes in transgenic lines were all higher than WT (Figure 7F–H), indicating that overexpression of *CabHLH79* lines had higher antioxidant stress resistance.

## 3. Discussion

BHLH TFs are a superfamily of plant-specific transcription factors, which play a vital role in plants to different harsh environments by regulating related stress genes. This study found that *CabHLH79* is a stress-responsive TF that plays a positive role in cold stress tolerance. RT-qPCR results showed that *CabHLH79* was consistently highly expressed under cold treatment, suggesting that *CabHLH79* may be involved in the cold stress response (Figure 2A). To validate our hypothesis and better understand the function of *CabHLH79*, we silenced *CabHLH79* in pepper and overexpression of *CabHLH79* in pepper or *Arabidopsis*. We found that silence of *CabHLH79* in pepper decreased its tolerance to cold stress, while transient overexpression of *CabHLH79* in pepper enhanced the cold resistance of plants. The *CabHLH79* transgenic *Arabidopsis* also had better growth status compared to WT under cold stress conditions. Additionally, quite a few stress-responsive genes (*AtRD29A*, *AtERD15*, and *AtCBF1*) were significantly up-regulated in *CabHLH79* transgenic plants compared to WT under low temperature stress. These results further illustrate the importance of *CabHLH79* in cold-responsive stress.

When plants are subjected to abiotic stress, a large number of ROS will be produced in plants, the excessive accumulation of toxic ROS will cause damage to various components in plants [46]. Therefore, elimination and reduction of ROS levels are essential for maintaining cellular homeostasis [47]. It is well known that otherwise the toxic influence of plant cells, ROS as a signal factor plays a vital role in the regulation of plant responses to various abiotic stress [48]. In order to maintain the stability of ROS levels in plants and reduce the damage caused by oxidative stress to plants, antioxidant enzymes play a key role in removing excess ROS [49]. Under cold conditions, SOD, POD, and CAT activities of gene-silenced *CabHLH79* plants were lower than those of TRV2 plants (Figure 3C–E), indicating that the silencing of the *CabHLH79* gene led to the declination of eliminate levels for ROS, and then causes to more fearful membrane damage. However, the *CabHLH79* transgenic *Arabidopsis* showed higher SOD, POD, and CAT activity than the WT (Figure 7C–E).And, *CabHLH79* transgenic plants enhanced the ability of ROS scavenging antioxidant enzymes to maintain cellular homeostasis. These results suggest that *CabHLH79* improves cold tolerance, in part due to its superior ROS scavenging system.

MDA and REL are related to the membrane system [5,50]. MDA content is a representative physiological index to evaluate plant stress tolerance, which can indicate the degree of cell damage [51]. Electrolyte leakage is also an important physiological indicator of membrane injury [52]. Thus, MDA content and electrolyte leakage were performed to analyze the function of *CabHLH79* overexpression in decreasing membrane injury under cold conditions. In this study, the MDA content and REL in control plants were higher than *CabHLH79* transgenic plants under cold stress. However, *CabHLH79*-silenced had higher MDA contents and REL. Collectively, these results show that *CabHLH79* positively regulates cold stress. In response to cold stress, the DAB and NBT staining showed that the *CabHLH79*-silenced plants had higher super-oxide radicals and H_2_O_2_ contents compared to control plants. On the contrary, *CabHLH79*-overexpressing plants showed lower super-oxide radicals and H_2_O_2_ contents than WT. The results showed that *CabHLH79* enhanced plant tolerance to oxidative stress, and thus improved the cold resistance of plants. Under cold stress, the expression of cold stress-related genes and antioxidant-related genes increased significantly in *CabHLH79* overexpressed plants (Figure 5). Notably, transient overexpression of *CabHLH79* induced transcription of cold-related genes *CaERD15*, *CaRD29A,* and *CaCBF1A*, suggesting that *CabHLH79* may regulate the expression of these genes to improve cold resistance in plants. However, the regulatory relationship between *CabHLH79* and its cold-related genes remains to be further studied.

In conclusion, *CabHLH79* enhanced cold resistance by regulating the expression of antioxidant system and cold-related genes. In this study, *CabHLH79* played as an upstream transcription regulator of *CaNAC035* in pepper. virus-induced gene silencing (VIGS)knockdown *CabHLH79* enhanced the sensitivity of plants to cold stress, and overexpression of *CabHLH79* enhanced cold tolerance of *Arabidopsis* and pepper. These findings demonstrated the cold resistance of *CabHLH79* from physiological and molecular aspects.

## 4. Materials and Methods

### 4.1. Yeast One-Hybrid (Y1H) Assays

The leaves of ‘P70’ pepper were used as materials exposed to cold treatment (4 °C) to construct a yeast one-hybrid cDNA library. The method of Y1H screening library was carried out according to the instructions of Matchmaker Gold Kit (Clontech, CA, USA). The truncated fragment of *CaNAC035* promoter (1–520 bp) was inserted into pAbAi vector then transform Y1H Gold to construct a yeast bait vector, which was used to screen cDNA library. After the bait yeast was grown on SD/-Leu^100^ (Aureobasidin A, 100 ng/mL) medium for 3–5 days, a single colony of yeast in a normal state was picked for PCR identification. The PCR products that showed positive were sequenced using T7 and 3’AD sequencing primers. After completion, the sequencing results of successful sequencing will be compared in the NCBI database for blast comparison.

For Y1H assays, the CDS region of the transcription factor *CabHLH79* was fused to pGADT7 to form a prey vector and transformed into the bait recombinant plasmid yeast strain containing the *CaNAC035* promoter. Positive yeast transformed cells were screened on selective SD/-Ura/AbA^200^ (Aureobasidin A, 200 ng/mL) plates. To determine the DNA-protein interaction, the yeast co-transformants were serially diluted (1:1, 1:10, 1:100, 1:1000) and cultured on SD/-Leu/AbA^200^ (Aureobasidin A, 200 ng/mL) deficient selection plates at 30 °C for 3–5 days.

### 4.2. Dual-Luciferase Assays

Dual-luciferase assay measured the transactivation effect of *CabHLH79* on the *CaNAC035* promoter. Using pepper DNA as a template, select restriction sites according to the vector sequence, design homologous recombination primers, amplify the 520bp promoter truncated sequence of *CaNAC035*, and inserted into the vector pGreenII0800-LUC containing restriction sites to generate the reporter plasmid proCaNAC035-LUC. Use the same method to select a suitable restriction site and insert the coding sequence of *CabHLH79* into pGreenII62-SK to form an effector plasmid CabHLH79-SK. Transform the recombinant plasmid into Agrobacterium GV3101. The pGreenII0800-LUC and pGreenII0800-CaNAC035-LUC vectors are used as reporter genes, the pGreenII62-SK and pGreenII62-CabHLH79-SK vectors are used as effector genes. Mix the Agrobacterium liquid containing Effector and Reporter in a ratio of 9:1, and inject the mixed bacterial liquid into the tobacco leaves. Three days after the injection, the leaves of different combinations were quickly ground into powder in liquid nitrogen and added to the cell lysate to mix for 5 min, centrifuged, and collected the supernatant. Use the Dual-Lucy Assay kit (Solarbio, Beijing, China) to detect the fluorescence activity according to the instructions.

### 4.3. Bioinformatics Analysis of CabHLH79

We obtained the full-length sequence of *CabHLH79* (Capana03g001053) through the pepper genome database (http://peppergenome.snu.ac.kr/) (accessed on 20 July 2020). Use online tools (http://web.expasy.org/compute_pi/) (accessed on 20 July 2020) to estimate the isoelectric point (pI) and molecular weight (Mw) of *CabHLH79*. Download the protein sequences of other crops from the GenBank database (https://www.ncbi.nlm.nih.gov/GenBank/) (accessed on 23 January 2021). In order to detect the phylogenetic relationship of bHLH, we used DNAMAN software to perform a multiple sequence alignment of *CabHLH79* protein to *Arabidopsis* bHLH protein. In the MEGA X software, a neighbor-joining (NJ) phylogenetic tree was constructed using JTT matrix-based model, 1000 bootstrap repeats.

### 4.4. Plant Materials and Growth Conditions

The WT *Arabidopsis thaliana* (Columbia ecotype), cultivar “P70” cold-tolerant strain pepper, and *Nicotiana benthamiand* were used throughout this study. All seeds were obtained from the College of Horticulture, Northwest A&F University, China. Both pepper plants and transgenic *Arabidopsis thaliana* were managed in an incubator with temperature conditions at a 22/18 °C (day/night) temperature cycle under 16h/8h (light/dark) long-day conditions, and the relative humidity is 75%.

### 4.5. RNA Extraction and RT-qPCR

Plant total RNA was extracted from 100 mg of young tissues of *Arabidopsis thaliana* and pepper. The RNA extraction method was following the instructions in the TianGen RNA extraction kit. (TianGen, Beijing, China). The synthetic method of single-stranded cDNA and RT-qPCR was as described by Chen et al. [53]. The pepper ubiquitin-binding gene *CaUbi3* (GenBank Accession No. AY486137.1), *Arabidopsis* Atactin gene (GenBank Accession No. AY572427.1) were used as internal reference genes for pepper and *Arabidopsis* [54]. The relative expression level of the gene was determined using the 2^-∆∆CT^ method [55]. The primers used in RT-qPCR were shown in Supplementary Table S1.

### 4.6. Virus-Induced Gene Silencing (VIGS) Assay of CabHLH79 in Pepper

To silence *CabHLH79*, use the website Sol Genomics Network (https://solgenomics.net/organism/Capsicum_annuum/genome) (accessed on 27 July 2020)to predict the specific region of the bHLH79 sequence, and select a 376 bp sequence in the specific region. Use specific primer forward F (F5′ GCTCTAGAAAGGGGCCAAGCTACTGAT 3′ XbaI) and reverse primer R (R5′ CGGGATCCTCATGTTGCTCTGTCAAAGCT 3′ BamHI) to amplify the *CabHLH79* fragment using pepper cDNA as a template and clone it into the pMD19T vector. The silent specific fragment was inserted into the pTRV2 vector containing the XbaI-BamHI site to form the pTRV2: *CabHLH79* recombinant plasmid. Subsequently, two-true-leaf stage peppers were injected according to the method of Wang et al. [56]. After about 30 days, when the leaves of the positive control plants appear to be chlorosis, plants were used for the silencing assay.

### 4.7. Transient Expression of CabHLH79 in Pepper Leaves

In order to the transient expression in pepper leaves, the recombination plasmid 35:CabHLH79-GFP and 35S:GFP were transformed into the Agrobacterium tumefaciens strain GV3101, the transient expression in pepper was followed by Cai et al. [57]. Transcriptional levels of *CabHLH79* in pepper leaves were detected by RT-qPCR.

### 4.8. Construction of Transgenic Arabidopsis Line Overexpressing CabHLH79

To construct the 35S:CabHLH79-GFP recombinant plasmid, the specific primer forward primer (F5′ TGCTCTAGA ATGGATCCACCTATTATTAATGAATC 3′XbaI) and reverse primer (R5′ CGGGGTACC TGTTGCTCTGTCAAAGCTGCT 3′KpnI) were used to amplify the *CabHLH79* coding region. The PCR amplified product was cloned into 35S:GFP vector. Use the freeze-thaw method to transfer the recombinant plasmid 35S:CabHLH79-GFP into Agrobacterium, and use the floral dipping methods to cultivate transgenic lines. The obtained seeds were screened in 1/2MS solid medium supplemented with 50 mg/L kanamycin to obtain transgenic plants, and T3 generation plants were harvested for subsequent use. In order to determine the successfully transgenic lines, DNA and RNA were extracted from the three-week-old transgenic *Arabidopsis* leaves of T3 generation [58]. The expression level of *CabHLH79* was detected via PCR and RT-qPCR, thereby two transgenic lines (OE3, OE15) were identified.

### 4.9. Cold Stress Tolerance Assays

To analyze the loss of function of *CabHLH79* pepper seedlings under cold stress, the *CabHLH79* silenced pepper plants were obtained by VIGS method. In order to explore the function of *CabHLH79* on cold stress in plants, transgenic Arabidopsis and transient overexpression of *CabHLH79* pepper were used as materials. For cold stress treatment, gene-silenced plants and overexpressed plants were treated at 4 °C for 3 days.

### 4.10. Biochemical Indices

The malondialdehyde (MDA) content was determined according to the method of Campos et al. [59] to estimate the amount of lipid peroxidation in the chloroplast membrane. To assess the permeability of the membrane, relative electrolyte leakage (REL) was detected as described by Danyluk et al. [60]. The total chlorophyll content of the extracted plant was calculated by spectrophotometry as described previously [61]. The activity of SOD, POD, and CAT were assayed following Dionisio-Sese and Jariteh et al. [62,63]. Using 3,30 diaminobenzidine (DAB) and nitro blue tetrazolium (NBT) staining to determine the accumulation of hydrogen peroxide (H_2_O_2_) and superoxide (O_2_^•−^) under cold stress [64].

### 4.11. Statistical Analysis

SPSS software was used for statistical analysis. Use the Mean ± SD (standard deviation) of three independent biological replicates to represent error bars. A one-way ANOVA test based on Fisher’s LSD test was used to calculate the significant difference analysis and significant differences relative to controls are indicated at * *p* < 0.05, ** *p* < 0.01, and *** *p* < 0.001.

## Data Availability

The date that support the results that included in this article and its supplementary materials. Other relevant materials are available from the corresponding author upon reasonable request.

## Supplementary Materials

The following supporting information can be downloaded at: https://www.mdpi.com/article/10.3390/ijms23052537/s1.

## References

[1] Thakur P., Kumar S., Malik J.A., Berger J.D., Nayyar H. (2010). Cold stress effects on reproductive development in grain crops: An overview. Environ. Exp. Bot..

[2] Thomashow M.F. (1999). Plant cold acclimation: Freezing tolerance genes and regulatory mechanisms. Annu. Rev. Plant Biol..

[3] Chinnusamy V., Zhu J., Zhu J.K. (2007). Cold stress regulation of gene expression in plants. Trends Plant Sci..

[4] Sanghera G.S., Wani S.H., Hussain W., Singh N.B. (2011). Engineering cold stress tolerance in crop plants. Curr. Genom..

[5] Huang X.S., Wang W., Zhang Q., Liu J.H. (2013). A basic helix–loop–helix transcription factor, PtrbHLH, of Poncirus trifoliata confers cold tolerance and modulates peroxidase-mediated scavenging of hydrogen peroxide. Plant Physiol..

[6] Zhu J.K. (2016). Abiotic stress signaling and responses in plants. Cell.

[7] Zandalinas S.I., Mittler R., Balfagón D., Arbona V., Gómez-Cadenas A. (2018). Plant adaptations to the combination of drought and high temperatures. Physiol. Plant..

[8] Lata C., Prasad M. (2011). Role of DREBs in regulation of abiotic stress responses in plants. J. Exp. Bot..

[9] Liu W., Tai H., Li S., Gao W., Zhao M., Xie C., Li W.X. (2014). bHLH122 is important for drought and osmotic stress resistance in Arabidopsis and in the repression of ABA catabolism. New Phytol..

[10] Li J., Li Y., Yin Z., Jiang J., Zhang M., Guo X., Ye Z., Zhao Y., Xiong H., Zhang Z. (2017). OsASR5 enhances drought tolerance through a stomatal closure pathway associated with ABA and H2O2 signalling in rice. Plant Biotechnol. J..

[11] Zhao C., Wang P., Si T., Hsu C.C., Wang L., Zayed O., Yu Z., Zhu Y., Dong J., Tao W.A. (2017). MAP kinase cascades regulate the cold response by modulating ICE1 protein stability. Dev. Cell.

[12] Chinnusamy V., Ohta M., Kanrar S., Lee B.H., Hong X., Agarwal M. (2003). ICE1: A regulator of cold-induced transcriptome and freezing tolerance in Arabidopsis. Genes Dev..

[13] Vogel J.T., Zarka D.G., Van Buskirk H.A., Fowler S.G., Thomashow M.F. (2005). Roles of the CBF2 and ZAT12 transcription factors in configuring the low temperature transcriptome of Arabidopsis. Plant J..

[14] Agarwal M., Hao Y., Kapoor A., Dong C.H., Fujii H., Zheng X. (2006). A R2R3 type MYB transcription factor is involved in the cold regulation of CBF genes and in acquired freezing tolerance. J. Biol. Chem..

[15] Yamaguchi-Shinozaki K., Shinozaki K. (2006). Transcriptional regulatory networks in cellular responses and tolerance to dehydration and cold stresses. Annu. Rev. Plant Biol..

[16] Puranik S., Sahu P.P., Srivastava P.S., Prasad M. (2012). NAC proteins: Regulation and role in stress tolerance. Trends Plant Sci..

[17] Shi Y., Tian S., Hou L., Huang X., Zhang X., Guo H., Yang S. (2012). Ethylene signaling negatively regulates freezing tolerance by repressing expression of CBF and type-A ARR genes in Arabidopsis. Plant Cell..

[18] Feller A., Machemer K., Braun E.L., Grotewold E. (2011). Evolutionary and comparative analysis of MYB and bHLH plant transcription factors. Plant J..

[19] Murre C., McCaw P.S., Baltimore D. (1989). A new DNA binding and dimerization motif in immunoglobulin enhancer binding, daughterless, MyoD, and myc proteins. Cell.

[20] Atchley W.R., Terhalle W., Dress A. (1999). Positional dependence, cliques, and predictive motifs in the bHLH protein domain. J. Mol. Evol..

[21] Carretero-Paulet L., Galstyan A., Roig-Villanova I., MartínezGarcía J.F., Bilbao-Castro J.R., Robertson D.L. (2010). Genome-wide classification and evolutionary analysis of the bHLH family of transcription factors in Arabidopsis, poplar, rice, moss, and algae. Plant Physiol..

[22] Li X., Duan X., Jiang H., Sun Y., Tang Y., Yuan Z., Guo J., Liang W., Chen L., Yin J. (2006). Genome-wide analysis of basic/helixloop-helix transcription factor family in rice and Arabidopsis. Plant Physiol..

[23] Niu X., Guan Y., Chen S., Li H. (2017). Genome-wide analysis of basic helix-loop-helix (bHLH) transcription factors in *Brachypodium distachyon*. BMC Genom..

[24] Wei K., Chen H. (2018). Comparative functional genomics analysis of bHLH gene family in rice, maize and wheat. BMC Plant Biol..

[25] Zhang T., Lv W., Zhang H., Ma L., Li P., Ge L., Li G. (2018). Genome-wide analysis of the basic Helix-Loop-Helix (bHLH) transcription factor family in maize. BMC Plant Biol..

[26] Ke Y.Z., Wu Y.W., Zhou H.J., Chen P., Wang M.M., Liu M.M., Li P.F., Yang J., Li J.N., Du H. (2020). Genome-wide survey of the bHLH super gene family in *Brassica napus*. BMC Plant Biol..

[27] Zhang Z., Chen J., Liang C., Liu F., Hou X., Zou X. (2020). Genome-wide identification and characterization of the bHLH transcription factor family in pepper (*Capsicum annuum* L.). Front. Genet..

[28] Zhao Q., Xiang X., Liu D., Yang A., Wang Y. (2018). Tobacco Transcription Factor NtbHLH123 Confers Tolerance to Cold Stress by Regulating the NtCBF Pathway and Reactive Oxygen Species Homeostasis. Front Plant Sci..

[29] Geng J., Wei T., Wang Y., Huang X., Liu J.-H. (2019). Overexpression of PtrbHLH, a Basic Helix-Loop-Helix Transcription Factor from *Poncirus trifoliata*, Confers Enhanced Cold Tolerance in Pummelo (*Citrus grandis*) by Modulation of H_2_O_2_ Level via Regulating a CAT Gene. Tree Physiol..

[30] Verma D., Jalmi S.K., Bhagat P.K., Verma N., Sinha A.K. (2020). A bHLH transcription factor, MYC2, imparts salt intolerance by regulating proline biosynthesis in Arabidopsis. FEBS J..

[31] Song Y., Li S., Sui Y., Zheng H., Han G., Sun X., Yang W., Wang H., Zhuang K., Kong F. (2022). SbbHLH85, a bHLH member, modulates resilience to salt stress by regulating root hair growth in sorghum. Theor. Appl. Genet..

[32] Yang T., Yao S., Hao L., Zhao Y., Lu W., Xiao K. (2016). Wheat bHLH-type transcription factor gene TabHLH1 is crucial in mediating osmotic stresses tolerance through modulating largely the ABA-associated pathway. Plant Cell Rep..

[33] Li F., Guo S., Zhao Y., Chen D., Chong K., Xu Y. (2010). Overexpression of a Homopeptide Repeat-Containing BHLH Protein Gene (OrbHLH001) from Dongxiang Wild Rice Confers Freezing and Salt Tolerance in Transgenic Arabidopsis. Plant Cell Rep..

[34] Li C., Yan C., Sun Q., Wang J., Yuan C., Mou Y., Shan S., Zhao X. (2021). The bHLH transcription factor AhbHLH112 improves the drought tolerance of peanut. BMC Plant Biol..

[35] Qiu J.R., Huang Z., Xiang X.Y., Xu W.X., Wang J.T., Chen J., Song L., Xiao Y., Li X., Ma J. (2020). MfbHLH38, a *Myrothamnus flabellifolia* bHLH transcription factor, confers tolerance to drought and salinity stresses in Arabidopsis. BMC Plant Biol..

[36] Zhou J., Li F., Wang J., Ma Y., Chong K., Xu Y. (2009). Basic Helix-Loop-Helix Transcription Factor from Wild Rice (OrbHLH2) Improves Tolerance to Salt- and Osmotic Stress in Arabidopsis. J. Plant Physiol..

[37] Liu H., Yang Y., Liu D., Wang X., Zhang L. (2020). Transcription Factor TabHLH49 Positively Regulates Dehydrin WZY2 Gene Expression and Enhances Drought Stress Tolerance in Wheat. BMC Plant Biol..

[38] An J., Wang X., Zhang X., Xu H., Bi S., You C., Hao Y. (2020). An Apple MYB Transcription Factor Regulates Cold Tolerance and Anthocyanin Accumulation and Undergoes MIEL1-mediated Degradation. Plant Biotechnol. J..

[39] Liang G., Zhang H., Li X., Ai Q., Yu D. (2017). BHLH Transcription Factor BHLH115 Regulates Iron Homeostasis in *Arabidopsis thaliana*. J. Exp. Bot..

[40] Meng F., Yang C., Cao J., Chen H., Pang J., Zhao Q., Wang Z., Qing Fu Z., Liu J. (2020). A BHLH Transcription Activator Regulates Defense Signaling by Nucleo-cytosolic Trafficking in Rice. J. Integr. Plant Biol..

[41] Waseem M., Li N., Su D., Chen J., Li Z. (2019). Overexpression of a Basic Helix–Loop–Helix Transcription Factor Gene, SlbHLH22, Promotes Early Flowering and Accelerates Fruit Ripening in Tomato (*Solanum lycopersicum* L.). Planta.

[42] Samarina L.S., Bobrovskikh A.V., Doroshkov A.V., Malyukova L.S., Matskiv A.O., Rakhmangulov R.S., Koninskaya N.G., Malyarovskaya V.I., Tong W., Xia E. (2020). Comparative Expression Analysis of Stress-Inducible Candidate Genes in Response to Cold and Drought in Tea Plant [*Camellia sinensis* (L.) Kuntze]. Front. Genet..

[43] Zhang H., Ma F., Wang X., Liu S., Saeed U.H., Hou X., Zhang Y., Luo D., Meng Y., Zhang W. (2020). Molecular and Functional Characterization of CaNAC035, an NAC Transcription Factor from Pepper (*Capsicum annuum* L.). Front. Plant Sci..

[44] Miller G., Suzuki N., Ciftci-Yilmaz S., Mittler R. (2010). Reactive oxygen species homeostasis and signalling during drought and salinity stresses. Plant Cell Environ..

[45] Zhang X., Wang W., Wang M., Zhang H.Y., Liu J.H. (2016). The miR396b of Poncirus trifoliata functions in cold tolerance by regulating ACC oxidase gene expression and modulating ethylene–polyamine homeostasis. Plant Cell Physiol..

[46] Mittler R. (2002). Oxidative stress, antioxidants and stress tolerance. Trends Plant Sci..

[47] Kiribuchi K., Jikumaru Y., Kaku H., Minami E., Hasegawa M., Kodama O., Seto H., Okada K., Nojiri H., Yamane H. (2005). Involvement of the Basic Helix-Loop-Helix Transcription Factor RERJ1 in Wounding and Drought Stress Responses in Rice Plants. Biosci. Biotechnol. Biochem..

[48] Baxter A., Mittler R., Suzuki N. (2014). ROS as key players in plant stress signalling. J. Exp. Bot..

[49] Suzuki N., Koussevitzky S., Mittler R.O.N., Miller G.A.D. (2012). ROS and redox signalling in the response of plants to abiotic stress. Plant Cell Environ..

[50] Mittler R., Vanderauwera S., Gollery M., Van Breusegem F. (2004). Reactive oxygen gene network of plants. Trends Plant Sci..

[51] Yu X., Liu Y., Wang S., Tao Y., Wang Z., Shu Y., Ma H. (2004). CarNAC4, a NAC-type chickpea transcription factor conferring enhanced drought and salt stress tolerances in Arabidopsis. Plant Cell Rep..

[52] Bajji M., Kinet J.M., Lutts S. (2002). The use of the electrolyte leakage method for assessing cell membrane stability as a water stress tolerance test in durum wheat. Plant Growth Regul..

[53] Chen R., Jing H., Guo W., Wang S.-B., Ma F., Pan B.-G., Gong Z.-H. (2015). Silencing of Dehydrin CaDHN1 Diminishes Tolerance to Multiple Abiotic Stresses in *Capsicum annuum* L. Plant Cell Rep..

[54] Gutierrez L., Mauriat M., Gunin S., Pelloux J., Lefebvre J.-F., Louvet R., Rusterucci C., Moritz T., Guerineau F., Bellini C. (2008). The Lack of a Systematic Validation of Reference Genes: A Serious Pitfall Undervalued in Reverse Transcription-Polymerase Chain Reaction (RT-PCR) Analysis in Plants. Plant Biotechnol. J..

[55] Livak K.J., Schmittgen T.D. (2001). Analysis of Relative Gene Expression Data Using Real-Time Quantitative PCR and the 2^−ΔΔCT^ Method. Methods.

[56] Wang J.-E., Liu K.-K., Li D.-W., Zhang Y.-L., Zhao Q., He Y.-M., Gong Z.-H. (2013). A Novel Peroxidase CanPOD Gene of Pepper Is Involved in Defense Responses to Phytophtora Capsici Infection as Well as Abiotic Stress Tolerance. Int. J. Mol. Sci..

[57] Cai W., Yang S., Wu R., Cao J., Shen L., Guan D., Shuilin H. (2021). Pepper NAC-type transcription factor NAC2c balances the trade-off between growth and defense responses. Plant Physiol..

[58] Clough S.J., Bent A.F. (1998). Floral Dip: A Simplified Method ForAgrobacterium-Mediated Transformation of Arabidopsis Thaliana: Floral Dip Transformation of Arabidopsis. Plant J..

[59] Campos P.S., Quartin V.N., Ramalho J.C., Nunes M.A. (2003). Electrolyte Leakage and Lipid Degradation Account for Cold Sensitivity in Leaves OfCoffea Sp. Plants. J. Plant Physiol..

[60] Danyluk J., Perron A., Houde M., Limin A., Fowler B., Benhamou N., Sarhan F. (1998). Accumulation of an Acidic Dehydrin in the Vicinity of the Plasma Membrane during Cold Acclimation of Wheat. Plant Cell.

[61] Arkus K.A.J., Cahoon E.B., Jez J.M. (2005). Mechanistic Analysis of Wheat Chlorophyllase. Arch. Biochem. Biophys..

[62] Dionisio-Sese M.L., Tobita S. (1998). Antioxidant Responses of Rice Seedlings to Salinity Stress. Plant Sci..

[63] Jariteh M., Ebrahimzadeh H., Niknam V., Mirmasoumi M., Vahdati K. (2015). Developmental Changes of Protein, Proline and Some Antioxidant Enzymes Activities in Somatic and Zygotic Embryos of Persian Walnut (*Juglans regia* L.). Plant Cell Tissue Organ Cult..

[64] Kim Y.-S., Kim S.-G., Park J.-E., Park H.-Y., Lim M.-H., Chua N.-H., Park C.-M. (2006). A Membrane-Bound NAC Transcription Factor Regulates Cell Division in Arabidopsis. Plant Cell.

