# Supplementary material for: CabHLH79 Acts Upstream of CaNAC035 to Regulate Cold Stress in Pepper
Source: Int J Mol Sci. 2022 Feb 25;23(5):2537. doi: 10.3390/ijms23052537 (PMC8910607; doi:10.3390/ijms23052537)
Supplement: Supplementary file 1 [file ijms-23-02537-s001.zip › ijms-1585925-supplementary.pdf]

Supplementary materials

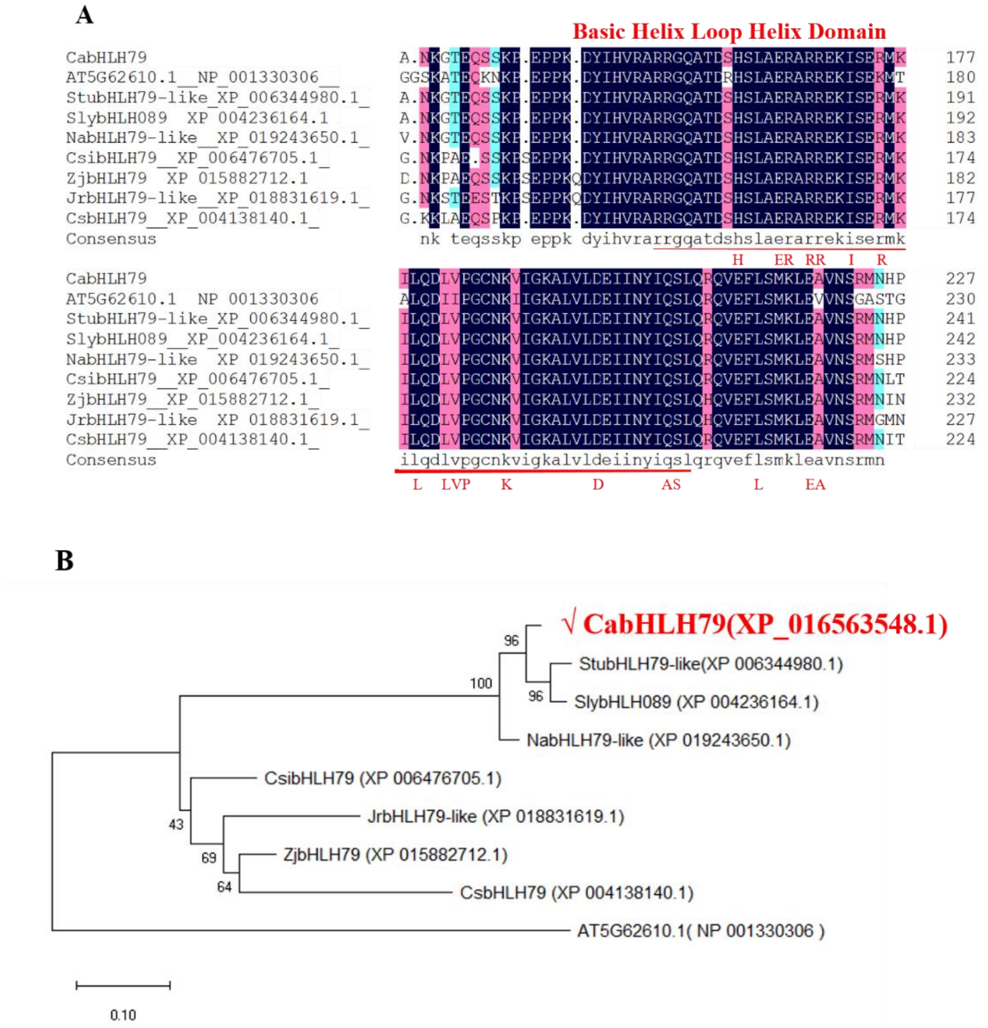

Figure S1. Sequence analysis of the CabHLH79 protein. **(A)** The sequence analysis of *CabHLH79*. The black areas represent homology level 100 %. The pink areas represent a level of homology greater than or equal to 75 %. The blue areas represent a level of homology greater than or equal to 50 %. The consensus subdomains in the bHLH binding domain are shown by thin underlines. **(B)** Phylogenetic relationship of *CabHLH79*(XP_ 016563548.1) and bHLH from other plant species. The protein sequence of CabHLH79 was used to perform the BLASTP search in the database NCBI Protein Reference Sequences (https://blast.ncbi.nlm.nih.gov/). Eight bHLH proteins were selected to construct the NJ phylogenetic tree by MEGA-X (http://www.megasoftware.net/) with the following parameters: JTT matrix-based model, bootstrap (1000 replicates), and pairwise deletion gaps.

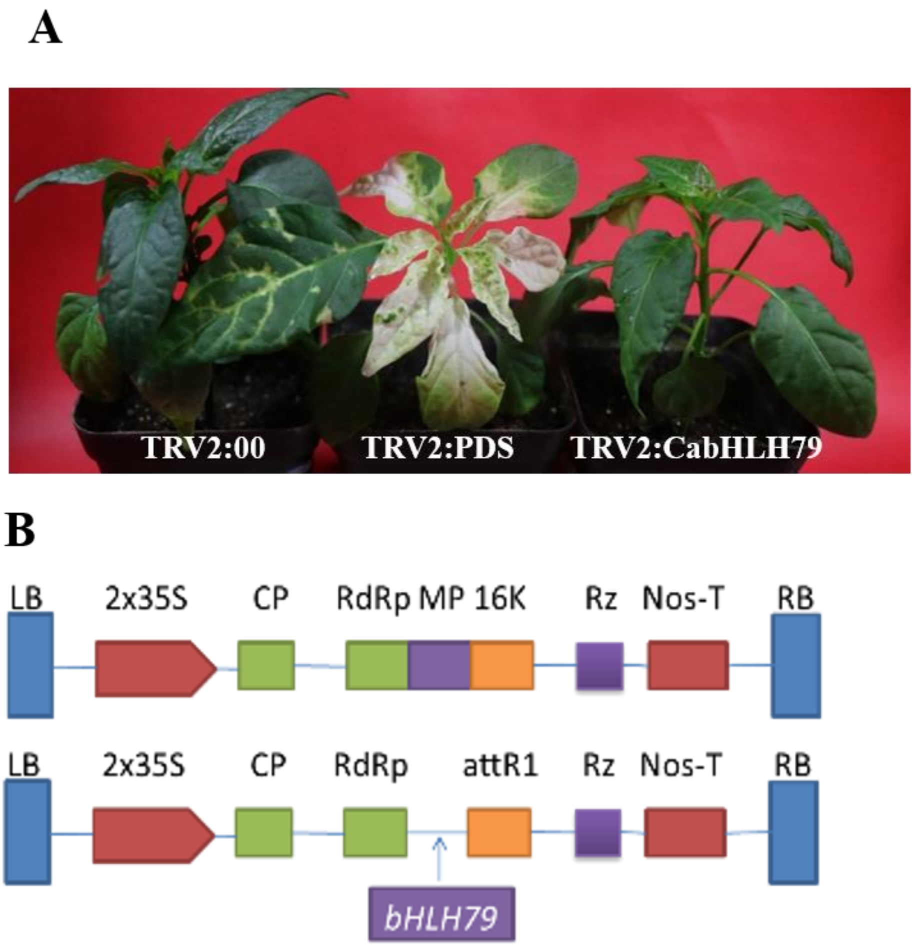

Figure S2. Phenotypes of plants with reduced expression of *CabHLH79*. **(A)** Phenotypes of pepper plants with reduced expression (about 4 weeks after injection). **(B)** Schematic diagram of the construction of VIGS vector. The experiment was conducted with three biological replicates and each replicate contained 3–5 pepper seedlings.

Table S1. Primers were used for the RT- qPCR.

Gene name	Primer Abbreviation	Primer Sequence (5'–3')
**1**	*CaUbi3-F*	TGTCCATCTGCTCTCTGTTG
**2**	*CaUbi3-R*	CACCCCAAGCACAATAAGAC
**3**	*CabHLH79-F*	CCAGCAGCTGTGTTCCACTA
**4**	*CabHLH79-R*	ACGAGACGACCATGAACATACA
**5**	*CaSOD-F*	TATGGAGCCTTAGAACCTGC
**6**	*CaSOD-R*	CCATTGAACTTGATAGCACCT
**7**	*CaPOD-F*	TCCTCCTCCTACTTCTAACC
**8**	*CaPOD-R*	ACAGACCTCTTTTGCTCACT
**9**	*CaCAT2-F*	GAAGCCAAATCCTAAGTCCC
**10**	*CaCAT2-R*	CCAACTCGGATTGCCTCTT
**11**	*CaAPX1-F*	AGAGGACAAGCCAGAACCAC
**12**	*CaAPX1-R*	CCTTGTCTGATGGCAACTGT

**13**	*ERD15-F*	CCAGCGAAATGGGGAAAC
**14**	*ERD15-R*	ACAAAGGTACAGTGGTGGC
**15**	*CaCBF1A-F*	TGAGGCTGTCGAGTCTTTC
**16**	*CaCBF1A-R*	AGGTGGAGGTAGCGTTAGT
**17**	*AtActin-F*	TGTTATGGTAGGGATGGGTC
**18**	*AtActin-R*	TTCTCTCTATTTGCCTTGGG
**19**	*AtCAT2-F*	GCAACTACCCCGAGTGGAAA
**20**	*AtCAT2-R*	TGTTCAGAACCAAGCGACCA
**21**	*AtPOD-F*	TCCGGGAGCCACACCATTGG
**22**	*AtPOD-R*	TGGTCGGAATTCAACAG
**23**	*AtSOD-F*	ATGAGAAGTTCTATGAAGAG
**24**	*AtSOD-R*	GTCTTTATGTAATCTGGT
**25**	*AtRD29A-F*	AACCACCACTCAACACACAC
**26**	*AtRD29A-R*	AGTCGCACCATTCTCATGATG
**27**	*AtKIN1-F*	GCGAAAGATCAAACTCCCCA
**28**	*AtKIN1-R*	TCCTTCTGTGTGTTTGGAAGA
**29**	*AtCBF1-F*	GTTTGGGATGCCGACTTTGTTGG
**30**	*AtCBF1-R*	GTCACCATCTCCTTCGCCGTCAT

Table S2. List of the 10 proteins selected following the Y1H screening.

Numbering	Gene ID	GenBank Accession
1	Capsicum annuum transcription factor MYC2-like (LOC107870881), mRNA	XM_016717573.1
2	Capsicum annuum ferredoxin-dependent glutamate synthase, chloroplastic (LOC107852264), mRNA	NM_001324694.1
3	Capsicum annuum transcription factor BIM1 (LOC107862352), transcript variant X2, mRNA	XM_016707907.1
4	**Capsicum annuum transcription factor bHLH79 (LOC107862470), mRNA**	**XM_016708062.1**
5	Capsicum annuum FGGY carbohydrate kinase domain-containing protein (LOC107877499), mRNA	XM_016724156.1
6	Capsicum annuum abscisic acid 8'-hydroxylase 1-like (LOC107850059), mRNA	XM_016694530.1
7	Capsicum annuum uncharacterized LOC107867496(LOC107867496), mRNA	XM_016713756.1
8	Capsicum annuum protein BRASSINAZOLE-RESISTANT 1-like (LOC107867043), mRNA	XM_016713134.1
9	Capsicum annuum phosphatidylinositol:ceramide inositolphosphotransferase 2-like (LOC107842870), mRNA	XM_016686913.1
10	Capsicum annuum transcription factor MYC2-like (LOC107850747), mRNA	XM_016695492.1
